# Opportunities and challenges of hole transport materials for high‐performance inverted hybrid‐perovskite solar cells

**DOI:** 10.1002/EXP.20220027

**Published:** 2023-02-24

**Authors:** Ru Li, Xue Liu, Jiangzhao Chen

**Affiliations:** ^1^ Key Laboratory of Optoelectronic Technology and Systems (Ministry of Education) College of Optoelectronic Engineering Chongqing University Chongqing China

**Keywords:** challenges, hole transport material, inverted perovskite solar cell, long‐term stability, non‐radiative recombination, opportunities

## Abstract

Inverted perovskite solar cells (inverted‐PSCs) have exhibited advantages of longer stability, less hysteresis, and lower fabrication temperature when compared to their regular counterparts, which are important for industry commercialization. Because of the great efforts that have been conducted in the past several years, the obtained efficiency of inverted‐PSCs has almost caught up with that of the regular ones, 25.0% versus 25.7%. In this perspective, the recent studies on the design of high‐performance inverted‐PSCs based on diverse hole transport materials, as well as device fabrication and characterization are first reviewed. After that, the authors moved on to the interface and additive engineering that were exploited to suppress the nonradiative recombination. Finally, the challenges and possible research pathways for facilitating the industrialization of inverted‐PSCs were envisaged.

## Graphical abstract

In this perspective, the recent advancements of inverted perovskite solar cells based on diverse hole transport materials, including polymer‐, copper‐, nickel‐based and self‐assembled monolayers and others, have been systematically reviewed. In addition, the current research issues and possible future directions for industry applications are given from the authors’ point of view.

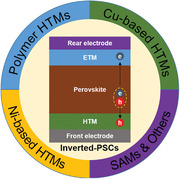

## 1 INTRODUCTION

**FIGURE 1:**
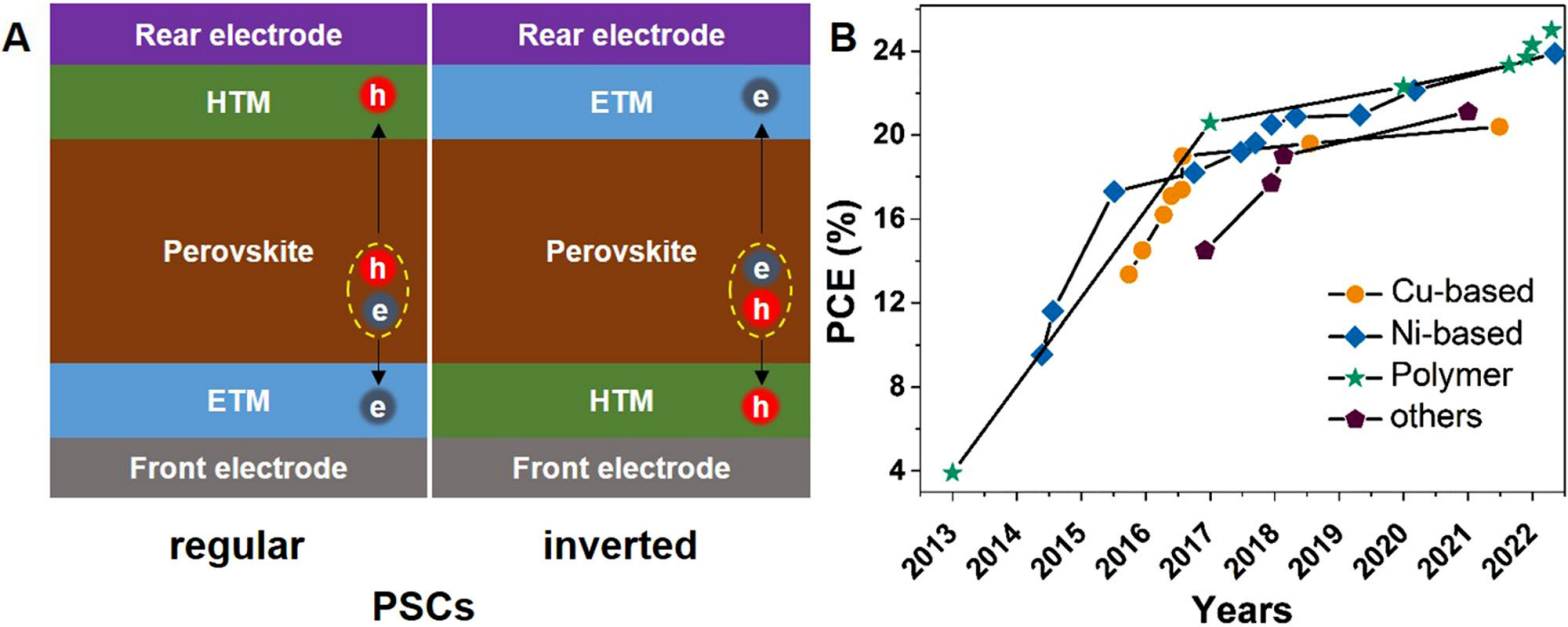
(A) Schematic of the regular‐ and inverted‐PSCs. (B) Efficiency evolution of inverted‐PSCs based on diverse HTMs.

Hydrogen nuclear fusion reaction has burned the sun for 4.5 billion years, whose radiation energy reaching the surface of the earth is around 100 mW cm^−2^. Scientists have predicted that the fusion reaction will continue for another 5 billion years, which is a long enough time for human beings. By rough calculation, only 1.1% of the territory is required to power China if it can be fully covered by solar cell panels. As a result, getting energy from the sun by solar cells becomes rather promising. Many photovoltaic technologies have been developed during the past several decades, among which the organic–inorganic hybrid perovskite solar cells (PSCs) have drawn much attention, whose power conversion efficiency (*PCE*) has been greatly improved from 3.8% in 2009 to a certified 25.7% in 2021 with a single‐junction device structure.^[^ ^1^ ^]^ However, the active area of high‐efficiency (>25%) PSCs is rather small (<0.1 cm^2^).^[^ ^2^ ^]^ Furthermore, the lifetime of PSCs is short (<2200 h), which is far behind the silicon solar cells (20–25 years). To obtain high‐efficiency, large‐area, and long‐lifetime PSCs for outdoor applications, the device structures, materials, and encapsulation are required to be fully optimized. According to the direction of current flow, the PSCs can be grouped into inverted (p‐i‐n) and regular (n‐i‐p) types (Figure 1A). For the regular PSCs, electrons are collected at the front transparent conducting electrode. For the inverted‐PSCs however, holes are collected instead. Although the regular PSCs currently have slightly higher efficiency, the inverted‐PSCs present advantages in longer stability, less hysteresis, and lower fabrication temperature that are favoured by industry applications.^[^ ^3^ ^]^

Central to the design of PSCs is the selection of electron and hole transport materials. For regular PSCs, organic hole transport material (HTM), typically the 2,2′,7,7′‐Tetrakis[*N*,*N*‐di(4‐methoxyphenyl)amino]‐9,9′‐spirobifluorene (Spiro‐OMeTAD) is employed. However, the necessary use of hygroscopic additives such as lithium bis(trifluoromethanesulfonyl)imide (Li‐TFSI) and 4‐tert‐butylpyridine (*t*BP) usually results in poor long‐term operational stability. In contrast to that, for the inverted‐PSCs, additive‐free hydrophobic [6,6]‐phenyl‐C_61_‐butyric acid methyl ester (PCBM) is used as electron transport material (ETM), the resultant inverted‐PSCs exhibit a longer lifetime due to the absence of hygroscopic additives. In addition, the use of PCBM is of specific interest due to its excellent electron conductivity and efficient passivation effect, which leads to negligible observed hysteresis.^[^ ^4^ ^]^ Figure 1B shows the efficiency evolution of inverted‐PSCs based on diverse HTMs, including polymer‐, copper‐, nickel‐based, and others. The structure design and photovoltaic parameters of inverted‐PSCs are summarized in Table 1. At this stage, the development of inverted‐PSCs slightly lags behind their regular counterparts. The first reported inverted‐PSC has a structural design of indium tin oxide (ITO)/poly(3,4‐ethylenedioxythiophene) polystyrene sulfonate (PEDOT:PSS)/CH_3_NH_3_PbI_3_ (MAPbI_3_)/PCBM/bathocuproine (BCP)/aluminium (Al) with an efficiency of only 3.9%, which did not gain wide attentions due to its poor performance.^[^ ^5^ ^]^ Only two years later, inverted‐PSCs with an efficiency of 15% and an active area as large as 1 cm^2^ were demonstrated,^[^ ^6^ ^]^ which then triggered extensive research on inverted‐PSCs.

**TABLE 1:** Photovoltaic parameters of inverted PSCs based on different HTMs.

HTMs	Device structures	*V_oc_* (V)	*J_sc_* (mA cm^−2^)	*FF*	*PCE* (%)	Area (cm^2^)	Year	ref
CuI	FTO/CuI/MAPbI_3_/PCBM/Al	1.040	21.06	0.62	13.58	NA	2015	^[^ ^22^ ^]^
CuI	FTO/CuI/MAPbI_3_/C_60_/PCBM/Al	1.010	22.80	0.73	16.8	0.1	2016	^[^ ^24^ ^]^
CuI	ITO/CuI/CuSCN/Perovskite/PC_61_BM/Ag	1.100	20.30	0.78	20.4	0.75	2021	^[^ ^28^ ^]^
Cu_2_O	ITO/Cu_2_O/MAPbI_3_/PC_61_BM/Ca/Al	1.070	16.52	0.76	13.35	0.04	2015	^[^ ^23^ ^]^
CuO*_x_*	ITO/CuO*_x_*/MAPbI_3_/C_60_/PCBM/Ag	0.990	23.20	0.74	17.1	0.1	2016	^[^ ^26^ ^]^
CuO*_x_*	ITO/CuO*_x_*/MAPbI_3‐_ *_x_* Cl*_x_*/PCBM/C_60_/BCP/Ag	1.110	22.50	0.76	19.00	0.1	2016	^[^ ^27^ ^]^
CuCrO_2_	ITO/CuCrO_2_/MAPbI_3_/PCBM/BCP/Ag	1.07	21.9	0.81	19.0	0.09	2018	^[^ ^32^ ^]^
Mg:CuCrO_2_	FTO/Mg:CuCrO_2_/MAPbI_3_/PCBM/BCP/Ag	1.00	18.4	0.713	13.1	0.06	2018	^[^ ^30^ ^]^
Mg:CuCrO_2_	ITO/Mg:CuCrO_2_/MAPbI_3_/C_60_/BCP/Ag	1.01	19.4	0.719	14.1	0.049	2019	^[^ ^31^ ^]^
CuAlO_2_	ITO/CuAlO_2_/PEDOT:PSS/MAPbI_3−_ *_x_* Cl*_x_*/PCBM/Ag	0.88	22.0	0.75	14.5	0.0725	2016	^[^ ^33^ ^]^
CuS	ITO/CuS/MAPbI_3_/C_60_/BCP/Ag	1.02	22.3	0.712	16.2	0.1	2016	^[^ ^25^ ^]^
NiO_x_/NiO_nc_	ITO/NiO*_x_*/NiO_nc_/MAPbI_3_/PCBM/BCP/Al	1.040	13.24	0.69	9.51	0.06	2014	^[^ ^35^ ^]^
sputtered‐NiO*_x_*/NiO_nc_	ITO/sputtered‐NiO*_x_*/NiO_nc/_MAPbI_3_/PCBM/BCP/Al	0.96	19.8	0.61	11.6	0.06	2014	^[^ ^36^ ^]^
Cu‐doped‐NiO*_x_*	ITO/Cu‐doped‐NiO*_x_*/Perovskite/PC_61_BM/C_60_/Ag	1.11	18.75	0.72	15.40	0.0314	2015	^[^ ^37^ ^]^
PLD‐NiO	ITO/PLD‐NiO/MAPbI_3_/PCBM/LiF/Ag	1.06	20.2	0.813	17.3	0.09	2015	^[^ ^41^ ^]^
NiMgLiO	FTO/NiMgLiO/MAPbI_3_/PCBM/Ti(Nb)Ox/Ag	1.072	20.62	0.748	16.2	1.02	2015	^[^ ^6^ ^]^
NiO	FTO/NiO/MAPbI_3_/gradient‐PCBM/Ti(Nb)O*_x_*/Ag	1.081	21.95	0.784	18.21	1.022	2016	^[^ ^38^ ^]^
NiO	FTO/NiO/MAPbI_3_/PCBM/Ag	1.122	23.17	0.757	19.19	1.025	2017	^[^ ^39^ ^]^
bl‐NiO*_x_*/mp‐NiO*_x_*	FTO/bl‐NiO*_x_*/mp‐NiO*_x_*/MAPbI_3_/PCBM/bis‐C_60_/Ag	1.11	21.58	0.819	19.62	0.0625	2017	^[^ ^40^ ^]^
Cu‐NiO*_x_*	FTO/Cu‐NiO*_x_*/MAPbI_3‐_ *_x_* Cl*_x_*/PC_61_BM/ZrAcac/Ag	1.12	23.7	0.7706	20.5	NA	2017	^[^ ^43^ ^]^
NiO*_x_*/mpCuGaO_2_	FTO/NiO*_x_*/mpCuGaO_2_/CsFAPb(I,Br)_3_/PC_61_BM/BCP/Ag	1.13	22.2	0.80	20	0.09	2018	^[^ ^42^ ^]^
F6TCNNQ/NiO*_x_*	ITO/F6TCNNQ/NiO*_x_*/mixed‐FAPbI_3_/PCBM/ZrAcac/Ag	1.12	23.18	0.803	20.86	0.12	2018	^[^ ^44^ ^]^
KCl‐NiO*_x_*	ITO/KCl‐NiO*_x_*/Perovskite/PCBM/ZrAcac/Ag	1.15	22.89	0.795	20.96	0.1	2019	^[^ ^45^ ^]^
NiO*_x_*/F2HCNQ	ITO/NiO*_x_*/F2HCNQ/PMMA/mixed‐Perovskite/PCBM/BCP/Ag	1.14	23.44	0.828	22.13	NA	2020	^[^ ^46^ ^]^
PEDOT:PSS	ITO/PEDOT:PSS/MAPbI_3_/C_60_/BCP/Al	0.60	10.32	0.63	3.9	0.06	2013	^[^ ^5^ ^]^
PTAA	ITO/PTAA/MAPbI_3_/Passivation layer/C_60_/BCP/Cu	1.1313	23	0.791	20.59	0.0716	2017	^[^ ^10^ ^]^
PTAA	ITO/PTAA/PFN‐P2/Perovskite/LiF/C_60_/BCP/Cu	1.103	23.2	0.784	20.1	1.013	2018	^[^ ^62^ ^]^
PTAA	ITO/PTAA/Perovskite/C_60_/BCP/Cu	1.1429	23.8	0.82	22.3	0.0669	2020	^[^ ^63^ ^]^
PTAA	ITO/PTAA/PEAI/Perovskite/PEAI/PCBM/BCP/Ag	1.162	24.13	0.846	23.7	0.045	2021	^[^ ^15^ ^]^
PTAA	ITO/PTAA/single‐crystal Perovskite/C_60_/BCP/Cu	1.10	26.2	0.79	22.8	NA	2021	^[^ ^14^ ^]^
PTAA	ITO/PTAA/Perovskite/PCBM/BCP/VO_2_/Ag	1.16	23.82	0.80	22.11	0.12	2021	^[^ ^20^ ^]^
PTAA	ITO/PTAA‐F4‐TCNQ/PMMA/mixed‐Perovskite/PBAI/PCBM/Bphen/Al	1.157	24.56	0.821	23.33	NA	2021	^[^ ^64^ ^]^
PTAA	ITO/PTAA/Perovskite/FcTc2/C_60_/BCP/Ag	1.184	25.68	0.8232	25.0	0.08	2022	^[^ ^19^ ^]^
P3CT‐N	ITO/P3CT‐N/mixed‐Perovskite/PbPyA2/PCBM/C_60_/TPBi/Cu	1.19	24.8	0.829	24.3	0.09	2022	^[^ ^18^ ^]^
CoO*_x_*	ITO/CoO*_x_*/MAPbI_3_/PCBM/Ag	20.3	0.95	0.755	14.5	0.09	2016	^[^ ^59^ ^]^
LiCoO_2_	ITO/LiCoO_2_/MAPbI_3_/C_60_/BCP/Ag	22.5	1.06	0.8	19.1	0.1	2018	^[^ ^60^ ^]^
Cu:CrO*_x_*	FTO/Cu:CrO*_x_*/MAPbI_3_/PCBM/BCP/Ag	1.08	21.4	0.76	17.7	0.09	2018	^[^ ^56^ ^]^
MoO*_x_*	ITO/MoO_3_/MAPbI_3_/PCBM/Ag	0.99	18.8	0.71	13.1	NA	2016	^[^ ^55^ ^]^
MoO*_x_*:RGO	ITO/MoO*_x_*:RGO/MAPbI_3_/PCBM/BCP/Ag	1.12	21	0.77	18.2	0.0177	2020	^[^ ^58^ ^]^
Cs:VO*_x_*	ITO/Cs:VO*_x_*/MAPbI_3_/PC_61_BM/BCP/Ag	0.92	20.7	0.765	14.5	0.057	2018	^[^ ^57^ ^]^
SAM	ITO/MC43/Perovskite/C_60_/BCP/Ag	1.08	20.1	0.8	17.3	NA	2019	^[^ ^50^ ^]^
SAM	ITO/EADR/mixed‐Perovskite/C_60_/BCP/Cu	1.156	22.9	0.8	21.20	NA	2021	^[^ ^51^ ^]^
SAM	ITO/N01(N02)/Perovskite/C_60_/BCP/Ag	1.15	23.67	0.8026	21.85	NA	2021	^[^ ^52^ ^]^
SAM	ITO/TPE‐S/CsFAPbI_3_/PCBM/ZnO/Ag	1.13	23.30	0.797	21.0	0.04	2020	^[^ ^53^ ^]^

For the majority of inverted‐PSCs, the ETMs are PCBMs or their derivatives, we thus focus on discussing the advancements of HTMs in this perspective. In this first part, the device structure, performance, advantages and disadvantages of inverted‐PSCs are systematically compared based on HTMs adopted. In the second part, we discuss the interface and defect passivation for minimizing nonradiative recombination losses. Finally, the urgent issues of inverted‐PSCs for commercial applications are analyzed.

## 2 HTMS IN INVERTED‐PSCS

**FIGURE 2:**
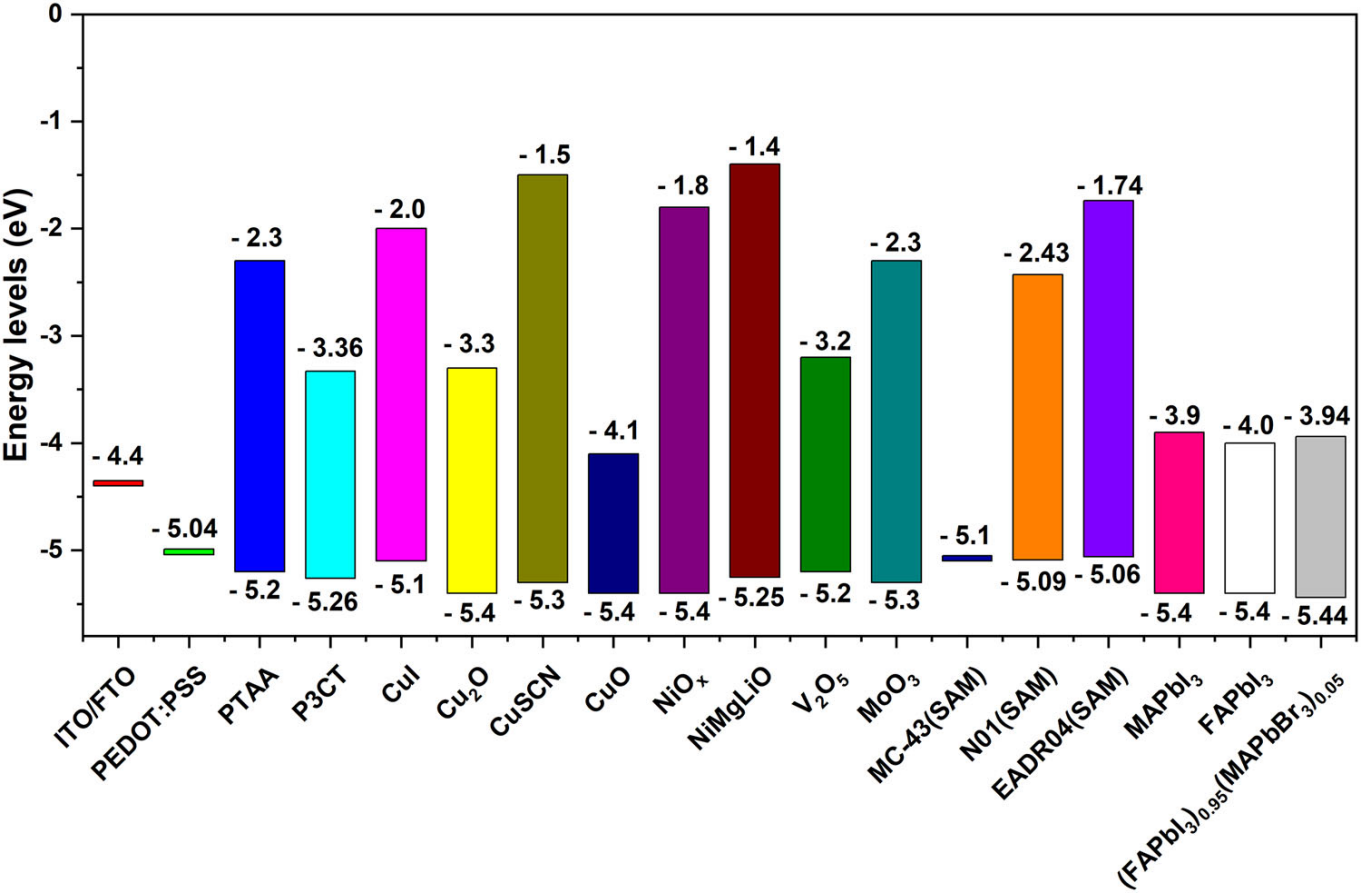
Energy level diagrams of commonly used HTMs in inverted‐PSCs.

Analysis of inverted‐PSCs begins from their device structure, which could be grouped into bulk heterojunction, graded bulk heterojunction, mesoporous and planar types. In the early days of inverted‐PSCs research, heterojunction and mesoporous structures were frequently used. However, planar structures are used nowadays due to their advantages in fabrication and performance. Given the crucial role of HTMs in inverted‐PSCs, we collected the energy levels of commonly used HTMs and compared them in Figure 2, energy levels of the most popular MA‐based, FA‐based and mixed‐perovskites were plotted as references.

### 2.1 Polymer HTMs

**FIGURE 3:**
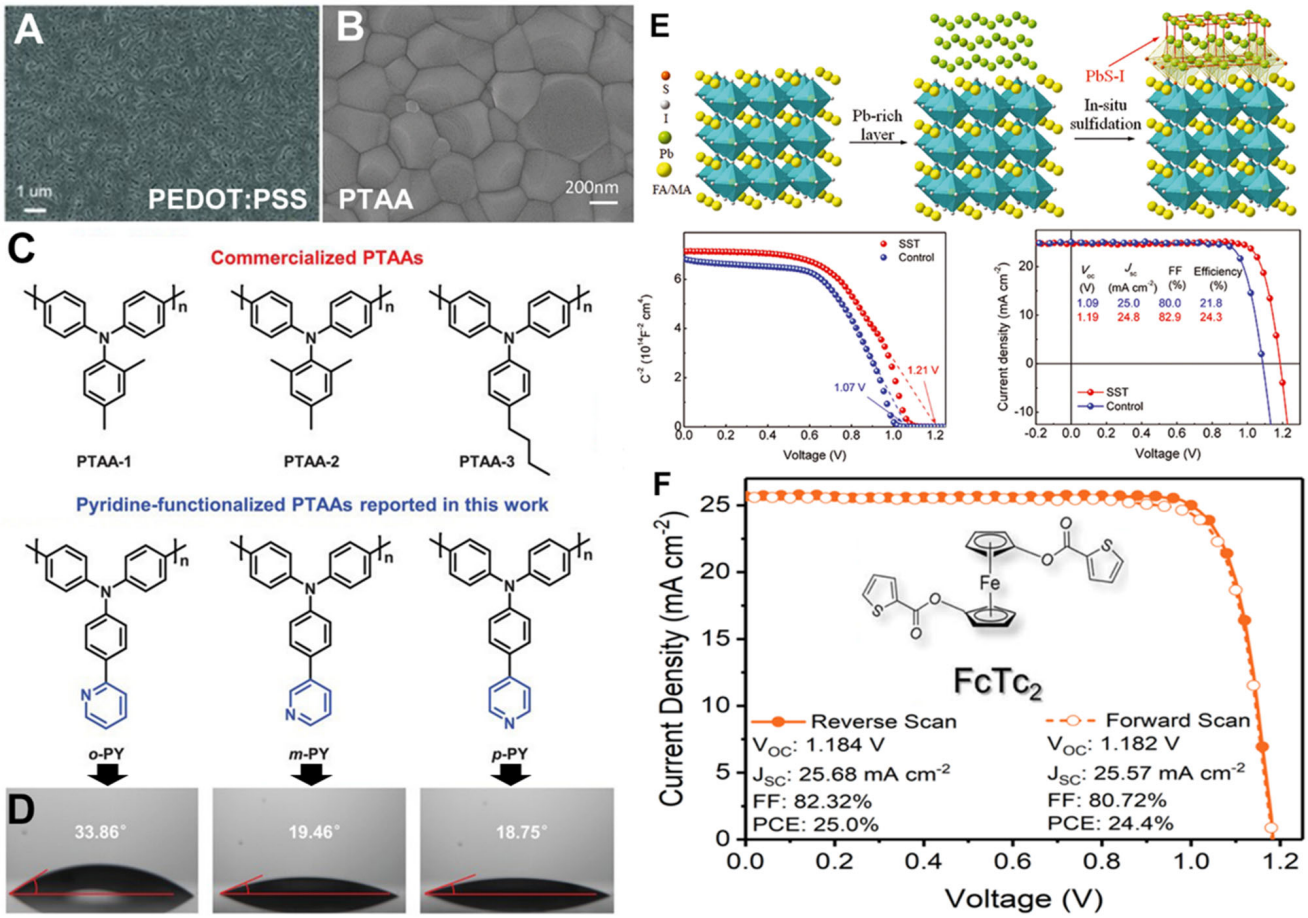
(A) SEM image of the perovskite fabricated on PEDOT:PSS. Reproduced with permission.^[^ ^5^ ^]^ Copyright 2013, Wiley‐VCH. (B) SEM image of the perovskite fabricated on PTAA. Reproduced with permission.^[^ ^10^ ^]^ Copyright 2021, Elsevier. (C) The Pyridine‐functionalized PTAAs with different linked sites. Reproduced with permission.^[^ ^11^ ^]^ Copyright 2021, Wiley‐VCH. (D) The contact angle of perovskite precursor solution on the Pyridine‐functionalized PTAAs. Reproduced with permission.^[^ ^11^ ^]^ Copyright 2021, Wiley‐VCH. (E) Surface sulfidation and the performance characterization of the inverted‐PSCs. Reproduced with permission.^[^ ^18^ ^]^ Copyright 2022, American Association for the Advancement of Science. (F) Inverted‐PSCs based on FcTc2 and the current‐voltage measurement. Reproduced with permission.^[^ ^19^ ^]^ Copyright 2022, American Association for the Advancement of Science.

The first reported inverted‐PSC was fabricated using PEDOT:PSS as HTM and delivered an efficiency of 3.9%. However, the hydrophilic and acidic characteristics of PEDOT:PSS limited the device's stability.^[^ ^5^ ^]^ Besides, the surface coverage of MAPbI_3_ onto which was poor, and tiny holes were observed (Figure 3A). To avoid the unfavourable hydrophilic feature, Alex et al. added a mild base imidazole to tune the pH value, they found that the crystallization of deposited perovskite was improved and the work function of PEDOT:PSS was changed from −4.91 to −5.31 eV, leading to a better energy level alignment. The improved energy level bending thus enabled a high open‐circuit voltage (*V_oc_*) of 1.06 V, much larger than that of the pristine PETOT:PSS 0.88 V, which delivered efficiency of 15.7%.^[^ ^7^ ^]^ Hydrophobic p‐type graphene oxide (GO) was found to have similar effects to that of the imidazole, the use of high conductivity GO doped PEDOT:PSS reduced the contact resistance, which led to an improved *PCE* from 15% to 18%.^[^ ^8^ ^]^ Beyond the hydrophobic additives, incorporating the metal oxides such as MoO*_x_*, GeO_2_ and NiO*_x_* into PEDOT:PSS was also found to be helpful in hole collection at the interface.^[^ ^9^ ^]^

Apart from the PEDOT:PSS, Huang et al. found that compact MAPbI_3_ could be obtained on poly[bis(4‐phenyl) (2,5,6‐trimethylphenyl) amine] (PTAA), as shown in Figure 3B, the average efficiency of 20.59% ± 0.45% was achieved.^[^ ^10^ ^]^ Although PTAA is promising as HTM, the hydrophobic nature, shallow highest occupied molecular orbital (HOMO) and absence of passivation groups often give rise to a low‐quality buried interface. Wu et al. thereby functionalized the PTAA using pyridine with varied linked sites, which are labelled as *o*‐PY, *m*‐PY and *p*‐PY in Figure 3C. It was found that the contact angle of the perovskite precursor solution decreased from 33.86° to 18.75° when the linked site is changed from ortho‐ to para‐position (Figure 3D). As the deep HOMO energy level of modified PTAA is favoured in band edge alignment, the pyridine‐modified PTAA exhibited a maximal *PCE* of 22%. High efficiency of ≈20% could be maintained even if the active area was increased to 1 cm^2^, which indicated a high uniformity of the PTAA layer.^[^ ^11^ ^]^ To overcome the hydrophobic nature of PTAA, Zhu et. al added the p‐type zwitterionic polysquaraine (PASQ‐IDT) into PTAA, they found that the wettability of perovskite precursor solution onto which is greatly improved, which led to high‐quality perovskite growth and translated into an efficiency of 21%.^[^ ^12^ ^]^ PASQ‐IDT can be used solely as HTM in inverted‐PSCs with an efficiency of 18.29%.^[^ ^13^ ^]^ To further improve the performance, mixed perovskite FA_0.6_MA_0.4_PbI_3_ with optimal bandgap was employed, which increased the short‐circuit current densities (*J*_sc_) to 26 mA cm^−2^ and maintained *V_oc_* of 1.10 V, resulting in an efficiency of 22.8%.^[^ ^14^ ^]^ It is commonly known that a large number of defects existed at both the top and buried surface of perovskite films, to address that, a dual interfacial modification strategy using large organic cations was proposed, such as (2‐phenylethyl ammonium iodide) (PEAI), which increased the *V*_oc_ and fill factor (*FF*) to 1.184 V and 0.85, respectively, yielding a *PCE* of 23.7%.^[^ ^15^ ^]^ Note that flexible PTAA‐based inverted‐PSCs could be fabricated if the rigid ITO is replaced by a flexible substrate, for example, the parylene‐VT4.^[^ ^16^ ^]^

Polyelectrolytes are another type of efficient HTMs for inverted‐PSCs. Fang et al. used the poly[3‐(4‐carboxybutyl)thiophene‐2,5‐diyl]‐Na (P3CT‐Na) as HTM to obtain an average efficiency of 15.4%.^[^ ^14^ ^]^ If the cation Na was replaced by K, Rb, Cs or CH_3_NH_3_^+^ cation, the efficiency could be increased to >20%.^[^ ^15^ ^]^ It should be noted that the P3CT‐Na had a strong aggregation tendency and unwell‐matched energy alignment, the diprophylline was then self‐assembled onto the ITO to modify the work function of the ITO, which then increased the P3CT‐Na inverted‐PSCs to 20.87%.^[^ ^17^ ^]^ In 2022, Fang et al. used the P3CT‐N as polymer HTM, mixed perovskite (FAPbI_3_)_0.95_(MAPbBr_3_)_0.05_ as light‐harvesting material, and surface sulfidation as Fermi level shifter, the resultant inverted‐PSCs showed a high *V*_oc_ of 1.19 V due to the extra back‐surface field induced by surface sulfidation. They obtained an efficiency of 24.3% for inverted‐PSCs,^[^ ^18^ ^]^ as shown in Figure 3E. Meanwhile, the surface sulfidation induced excellent operational stability more than 2200 h in an 85°C environment. Recently, Zhu et al. showed that organometallic molecular ferrocenyl‐bis‐thiophene‐2‐carboxylate (FcTc_2_) could improve the *J_sc_*, *V*_oc_ and *FF* of PTAA‐based inverted‐PSCs to 25.68 mA cm^−2^, 1.184 V and 0.8232, respectively, which set a record *PCE* of 25.0% (24.3% certified) for inverted‐PSCs,^[^ ^19^ ^]^ the structure of FcTc_2_ and device performance is shown in Figure 3F. For the PTAA‐based inverted‐PSCs, PCBM is the most studied ETM, Yang et.al showed that by adding a thin VO_2_ intermediate layer between PCBM and metal electrode, the electron extraction was enhanced, which led to an efficiency of 22.11%. If the temperature is increased to 85°C, the efficiency could be increased to 23% due to the phase change of VO_2_.^[^ ^20^ ^]^

The above results show that polymer HTMs are very prominent for inverted‐PSCs. However, the cost of polymer HTMs is rather expensive, around 200$ g^−1^, which is too high to be used in large‐scale industrial fabrication. Furthermore, polymer HTMs usually suffer from poor stability. More efforts should be devoted to the synthesis of low‐cost polymer HTMs, optimizing the molecular structures, understanding the correlation between molecular structures and their energy levels, surface contact angle of perovskite precursor solution onto which, the strain of perovskite layer grown on the HTMs,^[^ ^21^ ^]^ and subsequently the induced solar cell performance improvement.

### 2.2 Copper‐based HTMs

**FIGURE 4:**
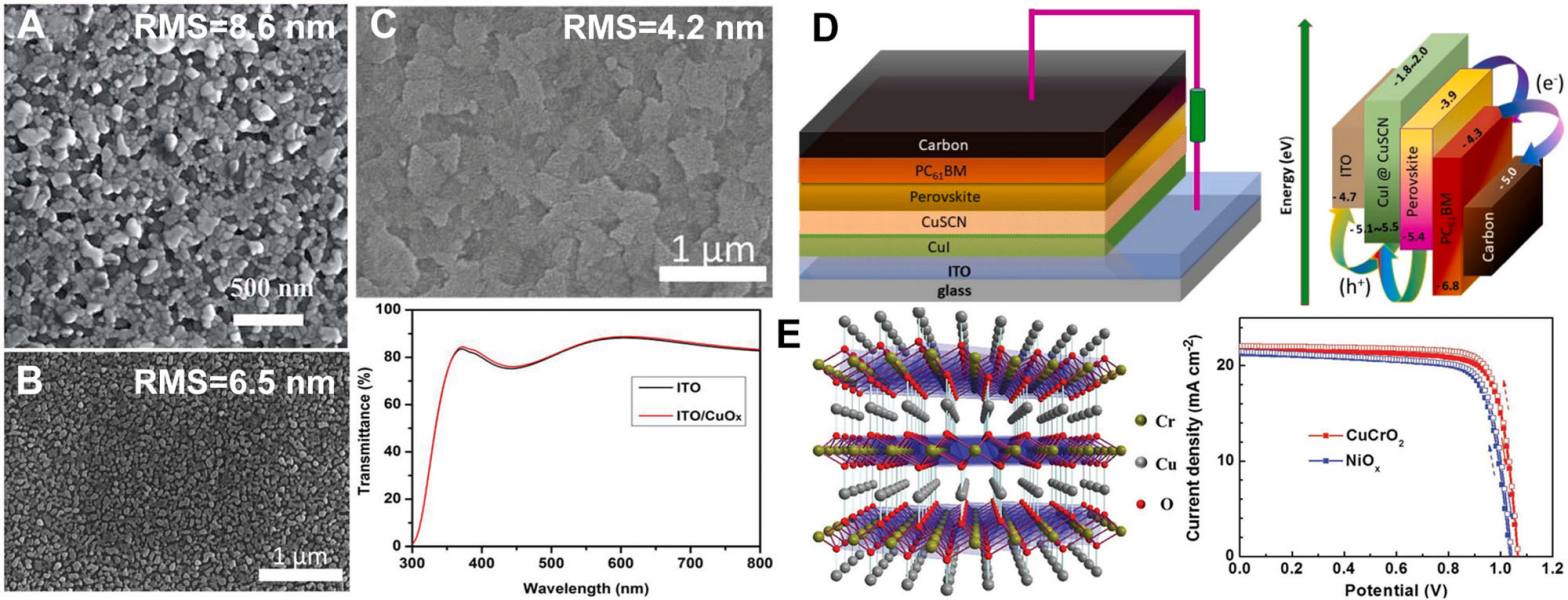
(A) SEM image of CuI with roughness of 8.6 nm. Reproduced with permission.^[^ ^22^ ^]^ Copyright 2015, Royal Society of Chemistry. (B) SEM image of CuI with roughness of 6.5 nm. Reproduced with permission.^[^ ^24^ ^]^ Copyright 2016, Royal Society of Chemistry. (C) SEM image of CuI with roughness of 4.2 nm and the corresponding transmittance. Reproduced with permission.^[^ ^26^ ^]^ Copyright 2016, Royal Society of Chemistry. (D) Inverted‐PSCs based on CuI@CusSCN HTMs. Reproduced with permission.^[^ ^28^ ^]^ Copyright 2021, Elsevier. (E) The crystal structure of CrCuO2 and the corresponding cell performance. Reproduced with permission.^[^ ^32^ ^]^ Copyright 2018, Wiley‐VCH.

The copper‐based oxides, iodides and sulphides are p‐type semiconductors with a hole mobility of 0.1–100 cm^2^ V^−1^ s^−1^, such as CuO, Cu_2_O, CuI, and CuS to name a few, which can be used as HTMs in inverted‐PSCs. In 2015, the solution‐processed CuI was used to fabricate inverted‐PSCs with an efficiency of 13.58%.^[^ ^22^ ^]^ In the same year, both of the Cu_2_O and CuO were utilized as HTMs to obtain comparable performance to that of the CuI.^[^ ^23^ ^]^ Note that the early copper‐based inverted‐PSCs had low efficiency, and later it realized that the surface roughness might be the key issue. As shown in Figure 4A, the surface roughness of CuI is high, reaching 8.6 nm, which is not suitable for high‐quality perovskite growth. Huang et al. found that by using the CuI layer with a reduced roughness of 6.5 nm as substrate (Figure 4B), high‐quality perovskite could be deposited, which translated into an improved efficiency of 16.8%.^[^ ^24^ ^]^ Beyond the CuI, the solution‐processed CuS nanoparticles could also act as efficient HTM, it changed the work function of ITO from −4.9 to −5.1 eV without sacrificing surface roughness and transmittance, leading to an efficiency of 16.2%.^[^ ^25^ ^]^

Non‐stoichiometric CuO*_x_* is a good HTM candidate for inverted‐PSCs. Bian et al. obtained an efficiency of 17.1% by using solution‐processed CuO*_x_* as HTM and MAPbI_3_ as light‐absorbing material. The CuO*_x_* not only improved the smoothness of pristine ITO but also increased the light transmittance, as shown in Figure 4C.^[^ ^26^ ^]^ Later on, they found that the efficiency could be increased to 19% by incorporating MAPbI_3‐_ *_x_* Cl*_x_* as light‐absorbing material due to morphology and hole mobility improvement.^[^ ^27^ ^]^ In 2021, Karuppuchamy et al. developed a two‐step electrodeposition way to fabricate CuI@CuSCN as HTM, the device structure of the solar cell and the corresponding energy levels are shown in Figure 4D. They achieved an efficiency of 20.35% and a long lifetime of up to 1400 h with only 10% performance loss.^[^ ^28^ ^]^ In comparison, the solution‐processed CuI@CuSCN‐based inverted‐PSCs could only last 156 h.^[^ ^29^ ^]^

Copper‐based delafossite compounds can also be used in inverted‐PSCs. Shin et al. used the CuCrO_2_ particulate films as HTMs and got an efficiency of 13.1%.^[^ ^30^ ^]^ Magnesium doping (Mg:CuCrO_2_) could increase the efficiency to 14.1% because of increased work function.^[^ ^31^ ^]^ Alex. et.al then demonstrated the low‐temperature solution‐processed CuCrO_2_ could achieve an efficiency of 19.0% due to improved transmission. The crystal structure of CuCrO_2_ and the corresponding current‐voltage measurements are shown in Figure 4E. The CuCrO_2_ has a bandgap of 2.9 eV, which can absorb wide UV light but maintain a high transmission above 400 nm, it thus can function as a UV‐blocking layer to alleviate the instability issue of perovskite materials induced by UV light. Note that the thickness of the CuCrO_2_ layer had a marked impact on *J*_sc_ and *FF*, a thick layer > 45 nm is favourable.^[^ ^32^ ^]^ The amorphous CuAlO_2_ layer deposited by magnetron sputtering could only deliver an efficiency of 14.52%.^[^ ^33^ ^]^ For the copper‐based inverted‐PSCs, the *J_sc_* and *FF* are generally less than 23 mA cm^−2^ and 0.80, respectively, which are inferior to that of other HTMs based competitors, the conductivity, transmittance, and work function of copper‐based HTMs should be optimized to obtain high performance.

### 2.3 Nickel‐based HTMs

**FIGURE 5:**
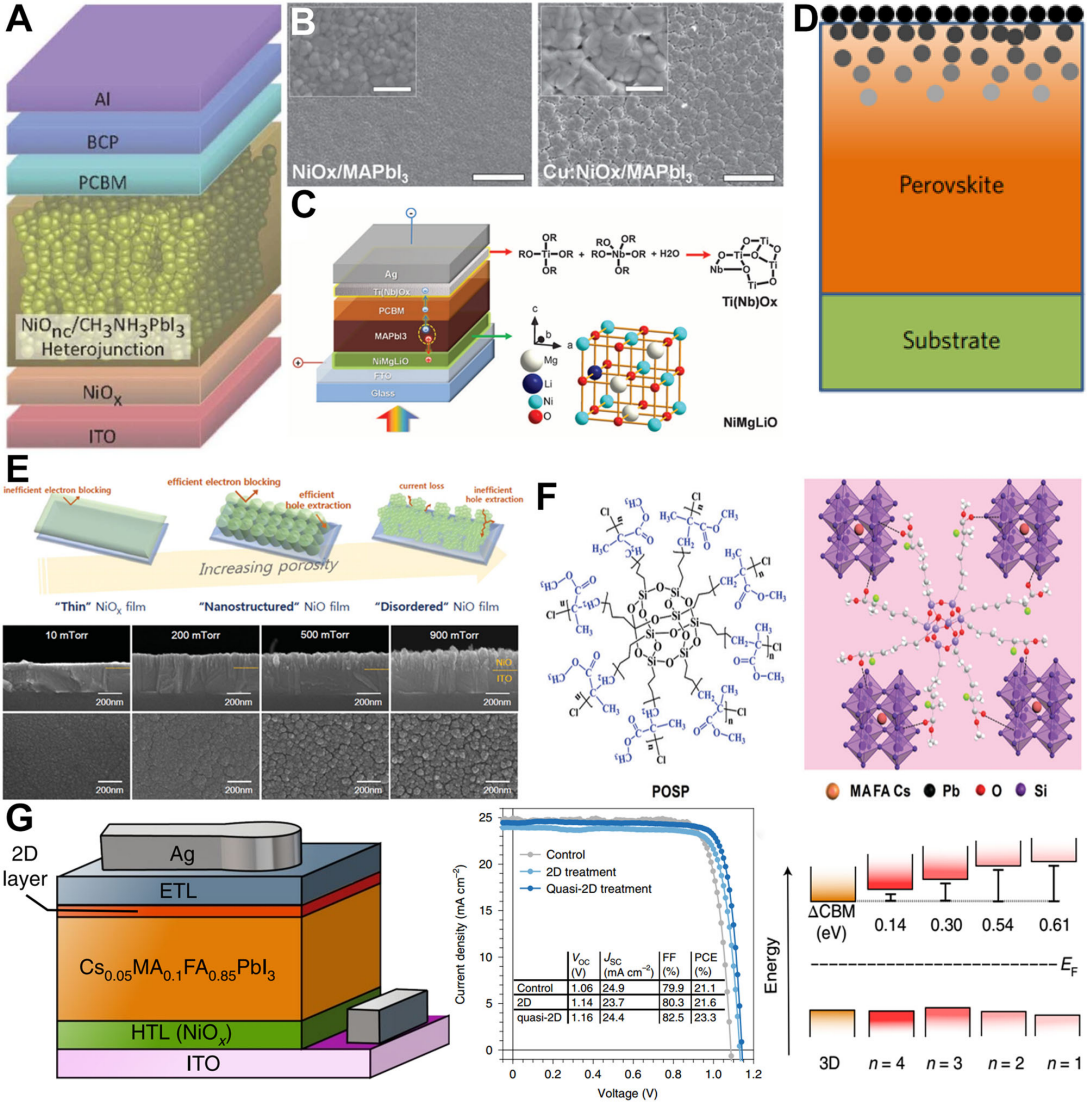
(A) NiO nanocrystal used in inverted‐PSCs. Reproduced under the terms of the CC BY‐NC‐SA 3.0 license. ^[^ ^35^ ^]^ Copyright 2014, Springer Nature. (B) SEM image of the perovskite on NiOx with and without Cu doping. Reproduced with permission.^[^ ^37^ ^]^ Copyright 2014, WILEY‐VCH. (C) Inverted‐PSCs based on NiMgLiO and Ti(Nb)Ox. Reproduced with permission.^[^ ^6^ ^]^ Copyright, 2015, American Association for the Advancement of Science. (D) The gradient heterojunction inverted‐PSCs based on NiMgLiO HTM. Reproduced with permission.^[^ ^38^ ^]^ Copyright 2016, Springer Nature. (E) Schematic of the nanostructured NiOx and the SEM images of NiOx prepared at different oxygen partial pressures. Reproduced with permission.^[^ ^41^ ^]^ Copyright 2015, WILEY‐VCH. (F) The chemical structure of the star polymer used to stabilize the perovskite. Reproduced with permission.^[^ ^47^ ^]^ Copyright 2021, Royal Society of Chemistry. (G) The 2D perovskite used in NiOx‐based inverted‐PSCs. Reproduced with permission.^[^ ^48^ ^]^ Copyright 2022, Springer Nature.

Among the inorganic materials that are suitable for inverted‐PSCs, NiO*_x_* has become an emergent alternative to copper‐based HTMs because of its high conductivity, matched work function, water resistance, and non‐corrosiveness. More importantly, NiO*_x_* films could be fabricated on rigid and flexible substrates by diverse techniques, such as spin‐coating, thermal evaporation, magnetron sputtering and atomic layer deposition.^[^ ^34^ ^]^ NiO*_x_* and NiO nanocrystals were firstly used by Guo et al. to construct heterojunction inverted‐PSCs, their device design is shown in Figure 5A. The device delivered an efficiency of only 9.51%.^[^ ^35^ ^]^ Just one year later, they used sputtered oxygen‐doped NiO*_x_* thin film as HTM, an efficiency of 11.6% was obtained due to improved optical transmission of NiO*_x_*.^[^ ^36^ ^]^ It was then found that copper‐doped NiO*_x_* (Cu:NiO*_x_*) was more efficient as a hole collection layer. The perovskite MAPbI_3_ on Cu:NiO*_x_* had a larger grain size when compared to pristine NiO*_x_* (Figure 5B), the resultant efficiency was increased to 15.4%.^[^ ^37^ ^]^ In 2016, Han et al. used inorganic NiMgLiO as hole collection layer and PCBM/Ti(Nb)O*_x_* as electron collection layer, they obtained an efficiency of 16.2% with an active area of 1.02 cm^2^, whose performance only dropped less than 10% after 1000 h under full sunlight illumination (Figure 5C).^[^ ^6^ ^]^ Han et al. then proposed a novel gradient perovskite–fullerene heterojunction device structure and showed that the gradient design could improve the electron collection and reduce the recombination loss for NiO*_x_*‐based inverted‐PSCs (Figure 5D). They achieved a *PCE* of 18.21% with an active area of 1.022 cm^2^. The devices exhibited a long lifetime of 1000 h under full light soaking.^[^ ^38^ ^]^ Soon after that, Han et al. showed that by incorporating methylammonium acetate thio‐semicarbazide into the MAPbI_3_ as additives, a large‐sized high‐quality perovskite layer could be obtained, resulting in certified efficiency of 19.19% with effective device size of 1.025 cm^2^.^[^ ^39^ ^]^ To minimize the NiO*_x_*/perovskite interface recombination loss, a mesoporous supported compact Cu:NiO*_x_* bilayer structure was proposed, which significantly improved stability and reduced the hysteresis, leading to an efficiency of 19.62%.^[^ ^40^ ^]^ By using a pulsed laser deposited nanostructured NiO*_x_* layer, Seok et al. showed that the efficiency of NiO*_x_*‐based inverted‐PSCs could be increased to 17.3%, the nanostructured NiO*_x_* layer not only blocked the electrons but also increased the porosity that was helpful for hole extraction, as presented in Figure 5D.^[^ ^41^ ^]^ Liu et.al demonstrated that a bilayered inorganic HTMs (compact NiO*_x_* and mesoporous CuGaO_2_) could improve the holes extraction, which then led to an efficiency of 20%.^[^ ^42^ ^]^

To further improve the performance, a systematic strategy including chlorine doping for MAPbI_3_, copper doping for NiO*_x_*, zirconium acetylacetonate modification for the aluminium cathode, and fluorine doping for ITO was applied, an efficiency of 20.5% was achieved for the inverted‐PSCs.^[^ ^43^ ^]^ Molecular doping is proved to be an efficient way to modulate the Fermi level of NiO*_x_*. The 2,2′‐(perfluoronaphthalene‐2,6‐diylidene)dimalononitrile (F6TCNNQ) was then employed, which decreased the Fermi level of NiO*_x_* from −4.63 to −5.07 eV, the gap between valence band maximum (VBM) of perovskite and Fermi level of NiO*_x_* was decreased from 0.58 to 0.29 eV. In doing so, the band mismatch between NiO*_x_* and perovskites was then lowered from 0.18 to 0.04 eV. As a result, the maximal efficiency of 20.86% was achieved due to the improved *V_oc_*.^[^ ^44^ ^]^ It was found that the alkali chloride interface modification of the NiO*_x_* interface could improve the ordering of perovskite, which pushed the *PCE* of CsFAMA‐based inverted‐PSC to 20.96%.^[^ ^45^ ^]^ A general rule of thumb to improve the *FF* of solar cells is to increase the conductivity of the NiO*_x_* layer, the 3,6‐difluoro‐2,5,7,7,8,8‐hexacyanoquinodimethane (F2HCNQ) was adopted and experimental results confirmed its capability to improve the conductivity of NiO*_x_* by five folds, which induced an efficiency of 22.13%.^[^ ^46^ ^]^ Beyond the small molecule, the polymer has been reported to have advantages in device stability, which motivates the exploration of novel polymer‐assisted high‐performance inverted‐PSCs. On account of this, Li et al. reported that an efficiency of 22.74% and operation stability as long as 1000 h could be obtained by incorporating a star‐polymer into the perovskite films. It is suggested that the inherited organic groups could directly chelate perovskite structures in multiple sites, which gives rise to excellent stability, the chemical structure of the star‐polymer is shown in Figure 5F.^[^ ^47^ ^]^ In 2022, Sargent et al. demonstrated that the NiO*_x_*‐based inverted‐PSCs could achieve a certificated efficiency of 23.9% by inserting a thin 2D perovskite between the PCBM and perovskite (Figure 5G). They revealed that the layer number (*n* ≥ 3) of the 2D perovskite was critical to obtaining high performance and long stability.^[^ ^48^ ^]^ Recently, our group proposed a precursor stabilization strategy to overcome the degradation of perovskite precursor solutions, the 3‐hydrazinobenzoic acid (3‐HBA) with functional groups of ─COOH and ─NHNH_2_ could effectively suppress the oxidation of I^−^, deprotonation of organic cations and amine‐cation reaction, which resulted in an efficiency of 23.3% for NiO*_x_*‐based inverted‐PSCs using the aged perovskite precursor solutions.^[^ ^49^ ^]^

The above results strongly imply that NiO*_x_* can act as efficient HTMs in inverted‐PSCs. Compared to the polymer HTMs, NiO*_x_* is cheaper and more stable, which is favoured by commercial applications. However, the efficiency of the published NiO*_x_*‐based inverted‐PSCs is lower than that of the polymer HTMs‐based competitors. Passivation of the oxygen vacancy defects of NiO*_x_*, fine‐tuning its work function and improving the conductivity should be conducted in the flowing studies.

### 2.4 Self‐assembled monolayers and others

**FIGURE 6:**
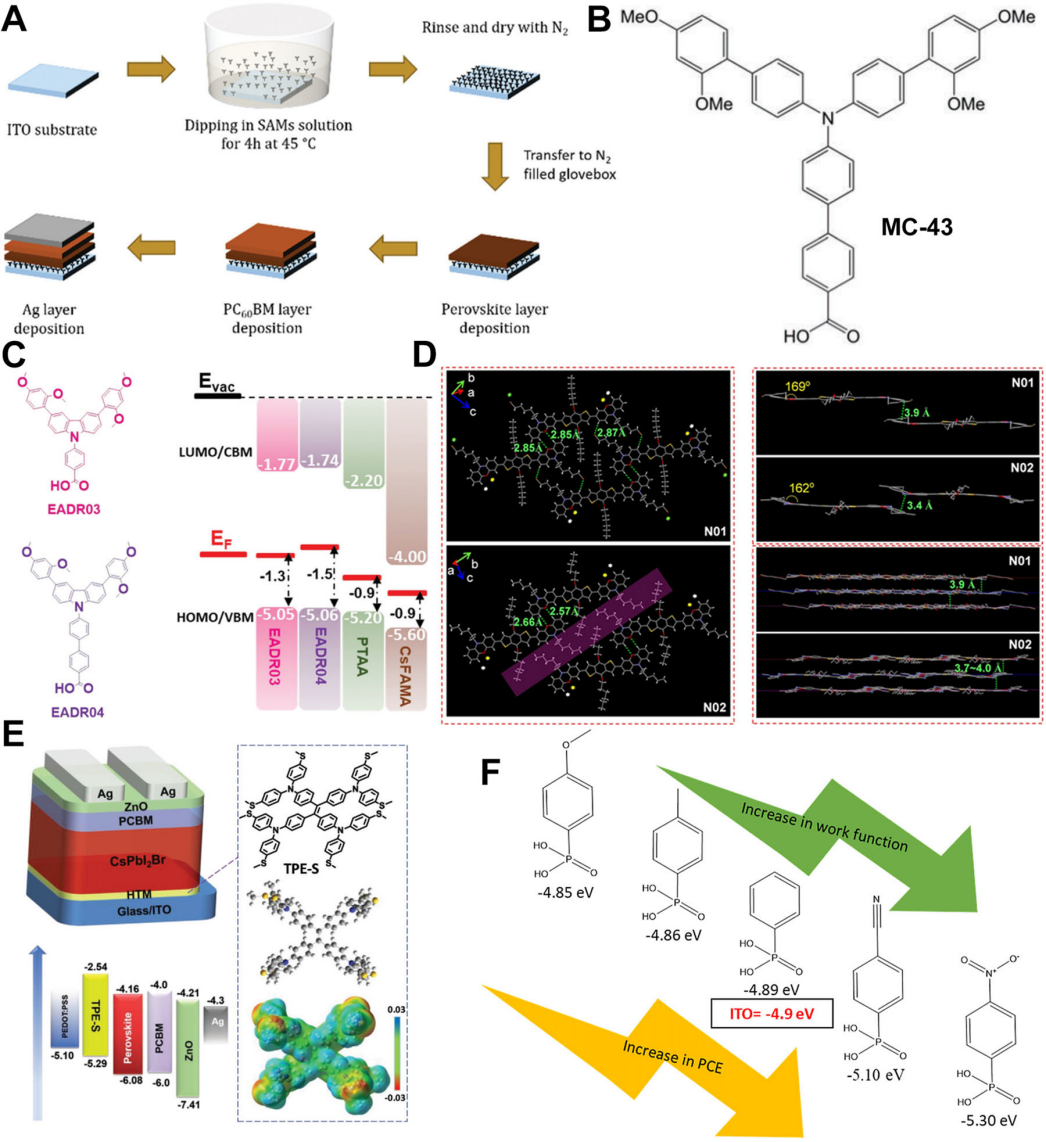
(A) Schematic of the general fabrication process for SAMs‐based inverted‐PSCs. Reproduced with permission.^[^ ^50^ ^]^ Copyright 2021, Royal Society of Chemistry. (B) The structure of MC‐43 SAMs. Reproduced with permission.^[^ ^50^ ^]^ Copyright 2021, Royal Society of Chemistry. (C) The chemical structure of EADR03 and EADR04 used in inverted‐PSCs. Reproduced with permission.^[^ ^51^ ^]^ Copyright 2021, Royal Society of Chemistry. (D) The chemical structure of N01 and N02 used in inverted‐PSCs. Reproduced with permission.^[^ ^52^ ^]^ Copyright 2021, Wiley‐VCH. (E) The TPE‐S based inverted‐PSCs, and the energy level diagram of the solar cells. Reproduced with permission.^[^ ^53^ ^]^ Copyright 2020, WILEY‐VCH. (F) The phenylphosphonic acids used to tune the work function of ITO. Reproduced with permission.^[^ ^54^ ^]^ Copyright 2021, Elsevier.

Recently, the self‐assembled monolayers (SAMs) have emerged as HTMs for inverted‐PSCs, they exhibit superiors in material consumption, bandgap modification, work function tuning and surface wetting enhancement, the general fabrication process of SAMs‐based solar cells is schematized in Figure 6A. Demic et al. used the 40‐[bis(20,40‐dimethoxybiphenyl‐4‐yl)amino]‐biphenyl‐4‐carboxylic acid (MC‐43) SAMs as an efficient hole transport layer, which led to an efficiency of 17.3%, where the chemical structure is shown in Figure 6B.^[^ ^50^ ^]^ Other SAMs, the 4‐(3,6‐bis(2,4‐dimethoxyphenyl)‐9H‐carbazol‐9‐yl)benzoic acid (EADR03) and 40‐(3,6‐bis(2,4‐dimethoxyphenyl)‐9H‐carbazol‐9‐yl)‐[1,10‐biphenyl]‐4‐carboxylic acid (EADR04) have been synthesized and utilized as HTMs, an efficiency of 21% has been obtained, the molecular structure and the device design are shown in Figure 6C.^[^ ^51^ ^]^ For the EADR SAMs‐based inverted‐PSCs, 80% of the initial performance could be maintained after 250 h of operation under the condition of AM 1.5 G light soaking and 85°C, which was still inferior to that of the other HTMs‐based inverted‐PSCs.^[^ ^51^ ^]^ Alex et al. recently reported an ordered SAMs lamellar structure, which was formed through in‐plane dipole‐dipole interaction and out‐plane π‐π interaction, as schematized in Figure 6D. The ordered conjugated network allows for delocalized electrons, and the hole collection and transportation is improved. What's more, high‐quality perovskite is easier to form on ordered SAMs layer, which gives rise to an efficiency of 21.85%.^[^ ^52^ ^]^ It is found that multi‐functional SAMs is more efficient, for example, Yip et. al replaced the oxygen (O) of tetraphenylethylene (TPE) with sulfur (S), named TPE‐S (Figure 6E), and demonstrated that the atom S could passivate defects of Pb^2+^ vacancies, which had an efficiency of 20%.^[^ ^53^ ^]^ Tao et.al used a series of para‐substituted phenylphosphonic acids to form SAMs on ITO, which modulated the work function of ITO gradually (Figure 6F), and a tamping method was employed to transfer the perovskite layer, which led to an efficiency of 13.94%.^[^ ^54^ ^]^ Although the SAMs have several advantages, however, the efficiency is generally less than 22%, which largely lags behind the polymer‐ and Ni‐based PSCs, the *J_sc_* is the limiting factor. The growth of high‐quality perovskite, as well as the electronic band match between SAMs and perovskite should be tuned.

Other hole conducting metal oxides, such as MoO*_x_*, WO*_x_*,^[^ ^55^ ^]^ Cu:CrO*_x_*,^[^ ^56^ ^]^ Cs‐doped VO*_x_*,^[^ ^57^ ^]^ MoO*_x_*:RGO,^[^ ^58^ ^]^ CoO*_x_*,^[^ ^59^ ^]^ and LiCoO_2_^[^ ^60^ ^]^ to name a few, have also been used as HTMs in inverted‐PSCs. The efficiencies of the devices based on those metal oxides lie in the range of 13.1–19.1%, whose performance could be further improved if the interface, composition of perovskites and fabrication process can be optimized.

From a personal viewpoint, the NiO*_x_* HTMs may be the best choice for the industrial application of inverted‐PSCs, because they are cheap, stable, and can be deposited in scalable ways. The passivation of the defects of NiO*_x_* is the leading factor to obtain high efficiency. We believe that the NiO*_x_*‐based inverted‐PSCs can compete with the best regular PSCs if systematic optimization can be carried out, such as perovskite bandgap tuning by composition engineering, interface engineering by 2D perovskite, defects passivation by small molecules, and so on.

## 3 SUPPRESSION STRATEGY OF NONRADIATIVE RECOMBINATION

Since the discovery of PSCs, its research undergoes three stages, high‐quality perovskite film growth in 2014–2016, charge transport modulation in 2016–2017, defects passivation and interface energy level alignment after 2018. Nonradiative recombination loss within the bulk and at the interface has become the leading factor that hinders further performance improvement.^[^ ^61^ ^]^ As such, defects passivation and interface modulation should be conducted to obtain high‐performance inverted‐PSCs, interface engineering and additive engineering are the two frequently adopted strategies.

### 3.1 Interface engineering

**FIGURE 7:**
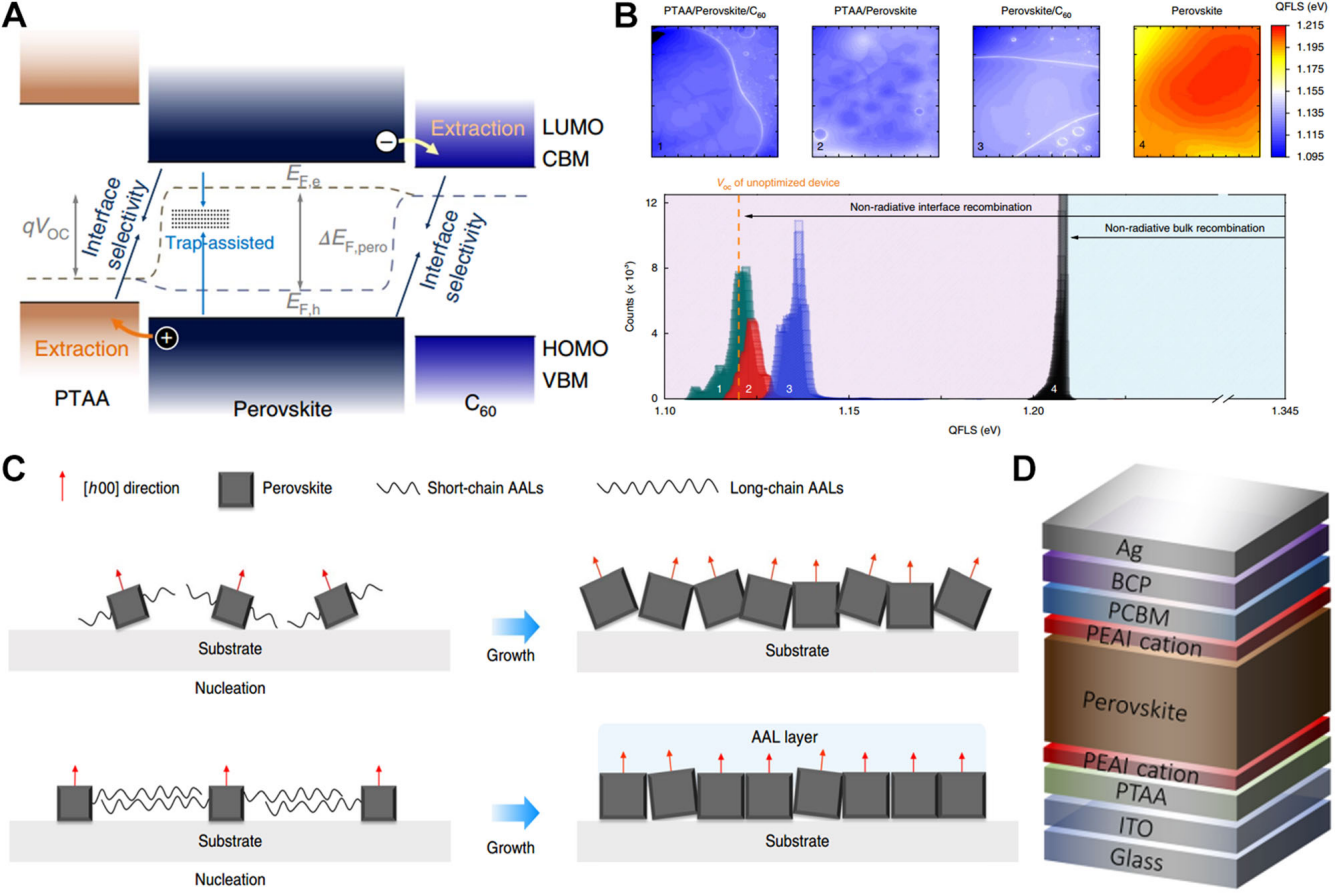
(A) Schematic energy diagram and pathway of non‐radiative energy loss. Reproduced with permission.^[^ ^62^ ^]^ Copyright 2018, Springer Nature. (B) Visualization of nonradiative interfacial recombination through absolute photoluminescence imagining. Reproduced with permission.^[^ ^62^ ^]^ Copyright 2018, Spring Nature. (C) Schematic of the mechanism underpinning efficiency enhancements by using long‐chain surfaceanchoring alkylamine ligand. Reproduced with permission.^[^ ^63^ ^]^ Copyright 2020, Spring Nature. (D) Solar cell structure with dual surface modification. Reproduced with permission.^[^ ^15^ ^]^ Copyright 2021, American Association for the Advancement of Science.

As shown in Figure 7A, both the trap‐induced recombination in bulk perovskite and the photo carrier recombination at the perovskite/transport layer interfaces account for the nonradiative loss. The nonradiative recombination pathways have been directly observed using transient and absolute photoluminescence mapping. It is found that a large quasi‐Fermi‐level splitting loss (≈135 meV) exists in the bulk perovskite, while a smaller interfacial loss (≈80 meV) is captured at the perovskite/PTAA and perovskite/C_60_ interfaces (Figure 7B), which limits the *V_oc_* to 1.12 eV. Ultrathin poly[(9,9‐bis(30‐((*N,N*‐dimethyl)‐*N*‐ethylammonium)‐propyl)‐2,7‐fluorene)‐alt‐2,7‐(9,9‐dioctylfluorene)] dibromide (PFN‐P2) and LiF are inserted into the perovskite/PTAA and perovskite/C_60_ interface, respectively, which effectively suppress the interface minority carrier recombination loss. The inverted‐PSCs show a *J_sc_* of 21.7 mA cm^−2^, *V_oc_* of 1.17 V, and *FF* of 81%, which is equivalent to an efficiency of 20%.^[^ ^62^ ^]^

Zheng et al. proposed that the surface‐anchoring alkylamine ligands (AALs) could be efficient grain and interface modifiers, and oriented perovskite growth was achieved by the long‐chain AALs, which lowered the trap density and enhanced the carrier mobilities (Figure 7C). They obtained an efficiency of 22.3% by adding AALs to CsFAMA mixed perovskite, the solar cells maintained the performance under 1000 h AM 1.5 light soaking.^[^ ^63^ ^]^ Degani et al. introduced PEAI to both the buried and top interface. It is widely accepted that PEAI would transform the 3D perovskite into 2D structures, however, they do not find any pieces of evidence of 2D perovskite formation may be due to the PEAI concentration introduced being small (Figure 7D). They observed an obvious PL intensity improvement, thus the passivation should be the leading effect, which led to an efficiency of 23.7% with improved *V_oc_* to 1.184 eV and *FF* to 85%, respectively.^[^ ^15^ ^]^ Zhang et al. used the same dual passivation strategy to both the top and buried surface of perovskite by polymethyl methacrylate (PMMA) and benzene butyl ammonium iodide (PBAI), respectively, an efficiency of 23.3% with long‐term stability against illumination and humidity were obtained, the trap density and ion migration were largely suppressed.^[^ ^64^ ^]^ Fang et.al used the pyridine‐2‐carboxylic lead (PbPyA2) for surface sulfidation and found that it was an efficient defect passivator, an efficiency of 24.3% for inverted‐PSCs was achieved.^[^ ^18^ ^]^ Recently, Zhu et.al used an organometallic chemical, ferrocenyl‐bis‐thiophene‐2‐carboxylate (FcTc_2_), to reduce the surface state trap density by strong Pb─O bonding. The charge transfer was accelerated through the delocalized ferrocene units, the as‐fabricated devices had a maximal *PCE* of 25.0% (certified 24.3%) and maintained more than 98% of their initial performance after 1500 h of standard 1‐sun light soaking. Moreover, the international standards for mature photovoltaics (IEC61215:2016) had been chosen to evaluate the devices' stability, the FcTc_2_ passivated devices passed the damp heat tests.^[^ ^19^ ^]^

Although the surface treatment benefits the defect passivation, it can lead to a negative effect, that is, unfavourable n‐type work function modulation, which facilitates the ion migration and therefore deteriorates the long‐term stability of PSCs.^[^ ^65^ ^]^ To obtain excellent surface/interface passivation, the following factors should be carefully balanced: (1) The positive side of defects passivation and negative side work function shift tradeoff should be comprehensively considered; (2) the possibilities of solvent corrosion during the spin‐coating process and the effects of high‐temperature annealing on the interlayer, (3) interlayer should be multifunctional, such as surface wettability modulation, energy band matching, and surface roughness trim.

### 3.2 Additive engineering

Apart from the perovskite/transport layer interface, additives have been added to bulk perovskites to reduce the trap density. It is well known that the PEDOS:PSS‐based inverted‐PSCs exhibit poor performance. The ionic liquid is introduced to passivate the uncoordinated Pb^2+^ of perovskite by its lone‐pair electron, such as the 1‐methyl‐3‐propylimidazolium bromide (MPIB), which increases the efficiency from 15.9% to 18.2% for PEDOT:PSS based inverted‐PSCs.^[^ ^66^ ^]^ Soon after that, Zhou et al showed that by adding 1‐Ethyl‐3‐methylimidazolium chloride (EMIC) into PEDOT:PSS and replacing PCBM with S‐acetylthiocholine chloride could efficiently reduce the charged defects in perovskite, which enhances the lifetime of photo carriers, leading to an efficiency of 20%.^[^ ^67^ ^]^

Alkali Chlorides have also been frequently used as additives in bulk perovskites, such as NaCl and KCl. Results show that they can improve the ordering of perovskite, which in turn reduces the defects and traps, leading to a big *V_oc_* improvement from 1.07 to 1.15 eV.^[^ ^45^ ^]^ Potassium thiocyanate (KSCN) is another inorganic salt used in bulk perovskite, which simultaneously links the NiO*_x_*, HTL and MAPbI_3_ together, leading to a *V_oc_* of 1.14 V and efficiency of 21.23%.^[^ ^68^ ^]^ Koo et al. added the conjugated small molecule bithiophene‐based (Y‐Th2) into perovskite and showed that defect states in perovskite could be effectively passivated through Lewis acid‐base interactions, an efficiency of 21.5% could then be obtained for PTAA‐based inverted‐PSCs.^[^ ^69^ ^]^ Carbon quantum dots are another efficient materials to passivate the surface and grain defects of perovskite, which could improve the efficiency and stability simultaneously.^[^ ^70^ ^]^ Li et.al added the biodegradability poly(butylene adipate‐coterephthalate) polymer (PBAT) into perovskite and found that the carbonyl groups and benzene rings could passivate the uncoordinated Pb^2+^ and neutral iodine defects of the perovskite, which led to an efficiency of 22.07% and long lifetime up to 3000 h.^[^ ^71^ ^]^

For the commonly used additives for bulk perovskite, although their content is rather small, for example, <5% weight, the effects on the crystalline dynamics of perovskite cannot be ignored. As such, more studies should be devoted to revealing the relationship between the molecular structure of additives and the crystal grain size of perovskite.

## 4 PERSPECTIVES

We would like to draw attention to the fact that the efficiency of inverted‐PSCs has reached 25.0% through the optimization of perovskite growth, energy level alignment, defects passivation, and interface modification. Furthermore, a mini‐module with a size of 57.2 cm^2^ has been fabricated, no performance degradation has been observed after 20 days of storage in an inert condition,^[^ ^72^ ^]^ indicating of bright future for industry application. However, the performance of inverted‐PSCs still lags a little behind their regular counterparts, the following suggestions are proposed to accelerate the research of inverted‐PSCs:

1. The field passivation materials are effective in Fermi energy shift and electron extraction, which has overcome the weakness of *V_oc_* of inverted‐PSCs. However, the uniformity of these materials on large scale is still challenging, large‐area field passivation should be developed in the future.
2. Compared to the regular PSCs counterparts, the performance of inverted‐PSCs is still slightly lower, further optimizing the electron transportation is required to improve the fill factor. Besides, increasing the transmission of the HTMs is also necessary to obtain a high short‐circuit current. Noting that the *V_oc_, J_sc,_* and *FF* are not independent parameters, they are strongly correlated with each other, therefore, the conductivity, transmission, and energy level of HTMs and ETMs should be carefully optimized.
3. PCBM is the most extensively used ETM in inverted‐PSCs, which is not only expensive but also lacks variations. Cheap non‐fullerene alternatives should be developed to meet the industry application, high conductivity and matched energy level are the two leading factors that should be considered for novel ETMs.
4. New chemically stable rear electrode materials, like carbon and Bi, have been developed for inverted‐PSCs, however, it is still challenging to get high conductivity at low temperatures, and more studies are needed in the future.

Currently, the urgent issue for inverted‐PSCs is to scale the high efficiency obtained on the small active area to a large module size. Besides that, improving the long‐term stability by advanced encapsulation methods should also be developed simultaneously.

## CONFLICT OF INTEREST STATEMENT

The authors declare no conflicts of interest.
